# Combinative treatment of Curdione and docetaxel triggers reactive oxygen species (ROS)-mediated intrinsic apoptosis of triple-negative breast cancer cells

**DOI:** 10.1080/21655979.2021.1994737

**Published:** 2021-12-02

**Authors:** Changcheng Wang, Jia Guo, Zeng’An Wu

**Affiliations:** Division of General Surgery, Wangjing Hospital, China Academy of Chinese Medical Sciences, Beijing, China

**Keywords:** Curdione, docetaxel, chemo-sensitization, reactive oxygen species (ROS), triple-negative breast cancer (TNBC)

## Abstract

Traditional Chinese medicine Curcuma zedoary has been used for treating various diseases and cancers. However, the therapeutic effect of Curdione, one of its major components in triple negative breast cancer (TNBC) is still obscure. This study is aimed to explore whether combination of Curdione and docetaxel (DTX) could strengthen the DTX-induced pro-apoptotic effects in TNBC cells and identify its involved signaling pathways. In this study, combination of Curdione and DTX intensified the inhibited MDA-MB-468 cell proliferation and increased cell apoptosis caused by DTX treatment alone. Moreover, the combinative treatment of Curdione and DTX synergistically potentiated DTX-induced cell apoptosis by triggering reactive oxygen species (ROS) generation. Co-treatment with NAC (ROS inhibitor) could mostly block the effects induced by combination of Curdione and DTX. SB203580 (p38 inhibitor) or SC-79 (Akt activator) could partly reverse the effects induced by co-treatment, indicating that mitogen-actived protein kinases (MAPKs) and the phosphatidylinositol 3-kinases (PI3K) /Akt signaling pathway were involved in the co-treatment induced ROS-mediated cell apoptosis. To sum up, combination of Curdione and DTX enhanced the chemotherapeutic efficacy on MDA-MB-468 cells by triggering ROS-mediated cell apoptosis via MAPKs and PI3K/Akt signaling pathways. Curdione combined with DTX might have potentials application as the therapeutic strategy for TNBC.

**Abbreviations:**

TNBC: triple negative breast cancer; ROS: reactive oxygen species; NAC: N-acetyl-L-cysteine; DTX: docetaxel; MAPKs: mitogen-actived protein kinases; PI3K/Akt: phosphatidylinositol 3-kinases (PI3K) /Akt; NF-Κb: the nuclear factor κB (NF-κB)

## Introduction

Triple-negative breast cancer (TNBC) is a specific subtype of breast cancer, which lacks the expressions of estrogen receptor (ER), progesterone receptor (PR) as well as human epidermal growth factor receptor 2 (HER-2) [1]. TNBC is characterized by high invasiveness and metastatic potential, and poor prognosis [2,3]. TNBC is classified into six subtypes by Lehmann et al., including basal-like 1 (BL1), basal-like 2 (BL2), mesenchymal (M), mesenchymal stem-like (MSL), immunomodulatory (IM), and luminal androgen receptor (LAR) [4]. Later, the authors refined these subtypes into four: basal-like 1 (BL1), basal-like 2 (BL2), mesenchymal (MES) and luminal androgen receptor (LAR) [5]. Each subtype has corresponding cell models. For example, the common cell lines MDA-MB-468, MDA-MB-231, and HCC1806 is defined as the basal-like 1 subtype, the mesenchymal stem-like subtype and the basal-like 2 subtype, respectively [1].

Chemotherapy is a successful and most important treatment for TNBC but often along with some adverse effects, which brings huge suffering to patients and their families [6]. Docetaxel is a first-line anti-tumor chemotherapy drug in clinical, mainly used for ovarian cancer, breast cancer and non-small cell lung cancer. Docetaxel, a specific drug that acts on the cell cycle, is cytotoxic to all the dividing cells as well as tumor cells. Undoubtedly, there are many side effects caused by docetaxel, including neutropenia, anemia, neuropathy, alopecia, and nail damage. It was observed that there were many undesirable side effects caused by weekly docetaxel treatment on epithelial ovarian cancer, such as fatigue, epiphora, nail changes and taste disturbances [7]. Recently, a case report found that docetaxel treatment may lead to myositis [8,9]. The side effects of docetaxel could not be ignored. It is reported that chemo-sensitization is an effective strategy to increase drug sensitivity and reduce side effects [10,11]. Therefore, it is an effective solution to develop more novel chemosensitizers to improve the effectiveness of chemotherapy in TNBC treatment.

*Curcuma zedoary* is a kind of traditional Chinese medicine which has been widely used for the gynecological diseases in China [12]. Phytochemicals derived from medicinal plants have been increasingly applied for diverse chronic diseases [13,14]. For instance, recent studies have revealed that the major components of Curcuma zedoary such as curcumin, curcumol have anti-inflammatory, antioxidant and anticancer properties in multiple diseases and cancers [15]. Moreover, recent studies reveal that traditional Chinese medicine compounds or extracts have advantages in enhancing the chemotherapeutic efficacy in combined chemotherapy with less harmful effects. For example, Formononetin could ameliorate the drug resistance of Taxol resistant TNBC [16]. Curcumol could enhance the sensitivity of doxorubicin in TNBC via regulating the miR-181b-2-3p/ABCC3 axis [17]. Melaleuca alternifolia essential oil and its main component terpinen-4-ol has great potential to improve target therapy response in melanoma [18]. However, as one of the major component of Curcuma zedoary, the enhanced chemotherapeutic efficacy and its molecular mechanisms of Curdione in TNBC still remains unclear.

In this study, we aimed to explore the efficacy of the combination treatment of Curdione and DTX on MDA-MB-468 and investigate the relevant signaling pathways. We hopefully provide the reference for the clinical treatment for TNBC.

## Methods

### Cell culture

Breast cancer cell line MDA-MB-468 (Cobioer, Nanjing, China) was cultured in Dulbecco’s Modified Eagle Medium (DMEM, Gibco; Thermo Fisher Scientific Inc., MA, USA) supplemented with 10% fetal bovine serum (FBS, Gibco, USA) and 1% penicillin-streptomycin (P/S, Solarbio Science & Technology Co., Ltd., Beijing) at 37°C in a humidified incubator with 5% CO_2_. Normal breast epithelial cell line MCF10A (Procell, Wuhan, China) was culture in DMEM/F12 (HyClone, Logan, UT, USA) with 10% FBS and 1% P/S. Cells were treated with different concentrations of Curdione and/or 1 µg/ml docetaxel (DTX, Sigma, MO, USA).

### Cell counting kit-8

Cell viability was determined by using Cell Counting Kit-8 (CCK-8, MCE, Shanghai, China). After treatment with different concentrations of Curdione, and/or 1 µM DTX, MDA-MB-468 cells were collected and seeded into the 24-well plate at the density of 5 × 10^4^ cells/well. After cultured for 24 h, 5 µL CCK-8 was added into each well and incubated at 37°C for 2 h. The absorbance was detected using a Microplate Reader (Bio-Rad, Hercules, CA, USA) at 450 nm.

### Western blot

The treated MDA-MB-468 cells were lysed with the cell lysis buffer. Protein concentration was measure by the BCA Protein Assay kit (Beyotime Biotechnolgy, Beijing). Lysates were separated in 10% SDS-PAGE and then transferred onto the nitrocellulose membrane (Millopore, USA). The membrane was blocked with 5% skimmed milk in TBST buffer at room temperature for 1 h. After that, the membranes were incubated with anti-Caspase 3 (1:5000, ab32351, Abcam, Cambridge, MA, USA), anti-Cleaved Caspase 3 (1:500, ab32042, Abcam), anti-Ki67 (1:5000, ab92742, Abcam), anti-PCNA (1:2000, ab92552, Abcam), anti-Bak (1:10000, ab32371, Abcam), anti-Bax (1:1000; ab32503, Abcam), anti-Bcl-2 (1:1000, ab32124, Abcam), anti-apaf-1 (1:1000, ab2001, Abcam), anti-Cytochrome c (1:5000; ab133504, Abcam), anti-Akt (1:1000; #4685, Cell Signaling Technology, Danvers, MA), anti-phosphorylated-Akt (1:1000, #4060 CST), anti-p38 (1:1000, #8690, CST), anti-phosphorylated p38 (1:1000, #4511, CST), anti-NF-κB (1:1000, #8242, CST), anti-p21 (1:1000; ab109520, Abcam), anti-p27 (1:5000; ab32034, Abcam) and GAPDH (1:2500, ab9485, Abcam) at 4°C overnight and then secondary antibodies (1:5000) were incubated for 1 h at room temperature. The proteins were visualized by using the enhanced chemiluminescence reagent (Bio-Rad, USA).

### Apoptosis assay

The apoptosis ratio was detected using the fluorescein isothiocyanate (FITC)-Annexin V apoptosis detection kit (BD Biosciences, San Jose, CA) according to the manufacturer’s protocol. Cells were treated with 70% precooling ethanol for 2 h then were incubated with propidium iodide (PI) and Annexin-V FITC. The apoptotic cells were analyzed using flow cytometry (FACScan; BD Biosciences).

### Intracellular ROS generation

The intracellular ROS generation was detected by flow cytometry using DCFH-DA (D6883, Sigma) according to the protocol. Cells were treated with 5 μM DCFH-DA at 37°C for 30 min and examined with the flow cytometer (BD Biosciences). The experiments were repeated for 3 times with each treatment.

### Statistics analysis

Each experiment was repeated at least three times. Data analysis was performed using GraphPad Prism 8 (GraphPad Software, Inc). Differences between groups were analyzed using the t test. All the data were presented as the mean ± standard deviation (SD).*p* < 0.05 is considered to be statistically significant.

## Results

In the present study, we evaluated the synergetic effect of Curdione and DTX and verified the relevant mechanism of the combination drugs on MDA-MB-468 cells. Firstly, we found that the combined treatment of Curdione and DTX intensified cell apoptosis of MDA-MB-468. Next, we revealed that co-treatment enhanced the DTX-induced cell apoptosis by inducing reactive oxygen species (ROS) level. Furthermore, we identified that combined treatment enhanced DTX-induced cell apoptosis via ROS-regulated MAPKs and PI3K/Akt signaling pathways in MDA-MB-468.

### Combinative treatment of Curdione and DTX enhances the inhibitory effects on cell proliferation of MDA-MB-468 induced by DTX

**Figure 1.**
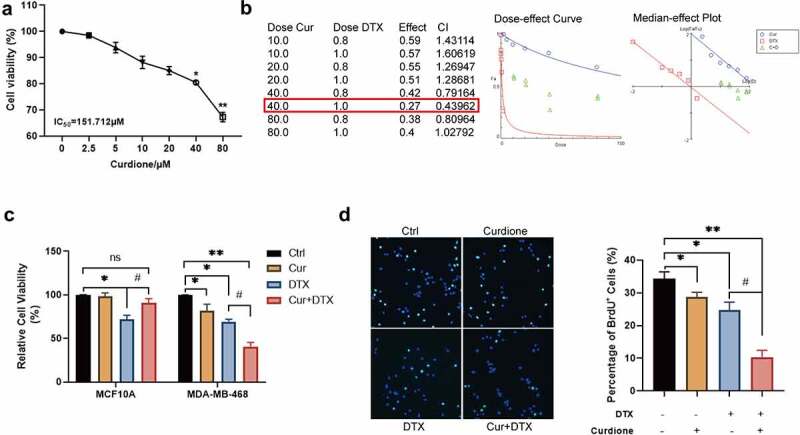
Curdione combined with DTX enhanced the inhibitory effects on cell proliferation in MDA-MB-468.(a) cell viability of MDA-MB-468 treated with different concentrations of Curdione (0, 2.5, 5, 10, 20, 40, 80 µM) for 48 h was performed by CCK-8. IC_50_ = 151.712 µM. * p < 0.05, ** p < 0.01. (b) The combination index (CI) of the combinative treatment of curdione and docetaxel on MDA-MB-468 was determined by Chou-Talalay method using CompuSyn software. the IC_50_ value of docetaxel for MDA-MB-468 was 0.911 µg/ml. the best synergetic effect of the combinative treatment of Curdione and docetaxel on MDA-MB-468 was gained from the treatment of 40 µM curdione with 1 µg/ml docetaxel for 48 h, of which CI is 0.43962. (c) the cell viability treated with Curdione (40 µM) and/or DTX (1 µg/ml) was measured in MDA-MB-468 and MCF10A cells by CCK-8. (d) BrdU staining was performed in MDA-MB-468 cells treated with Curdione (40 µM) and/or DTX (1 µg/ml). THE positive BrdU cells were calculated. *p < 0.05, **p < 0.01 versus the control group

To investigate whether the combination of Curdione and DTX could enhance the effect of DTX on basal-like 1 subtype TNBC cell line MDA-MB-468, firstly we assessed the effect of different concentrations of Curdione on MDA-MB-468 by CCK-8 (Figure 1(a)). We found that the inhibitory effect of Curdione on MDA-MB-468 cell proliferation was statistically significant starting from the concentration of 40 µM. The combination index (CI) of the combinative treatment of Curdione and DTX on MDA-MB-468 was determined by CompuSyn [19] as shown in (Figure 1(b)). When cells treated with 40 µM Curdione and 1 µg/ml DTX, CI was 0.43962 < 0.9, indicating the best synergistic effects of these two combined drugs. Moreover, CCK-8 analysis results showed that co-treatment of Curdione and DTX had an enhanced inhibitory effect on cell proliferation of MDA-MB-468 whereas Curdione or DTX alone had a slight inhibitory effect on MDA-MB-468 cell proliferation (Figure 1(c)). The cytotoxic effect of compounds used in monotherapy as well as their combination against normal cells MCF10A showed that Curdione had a protective effect on normal cell MCF10A when treated with DTX. Consistently, the BrdU staining results showed that the number of positive BrdU cells was much more decreased in co-treatment of Curdione and DTX compared to the DTX treatment alone. Taken together, these findings suggest that combinative treatment of Curdione and DTX has a raised effect on DTX-inhibited cell proliferation of TNBC cells.

### Treatment of Curdione and DTX potentiates the pro-apoptotic effects of DTX in TNBC cells

**Figure 2.**
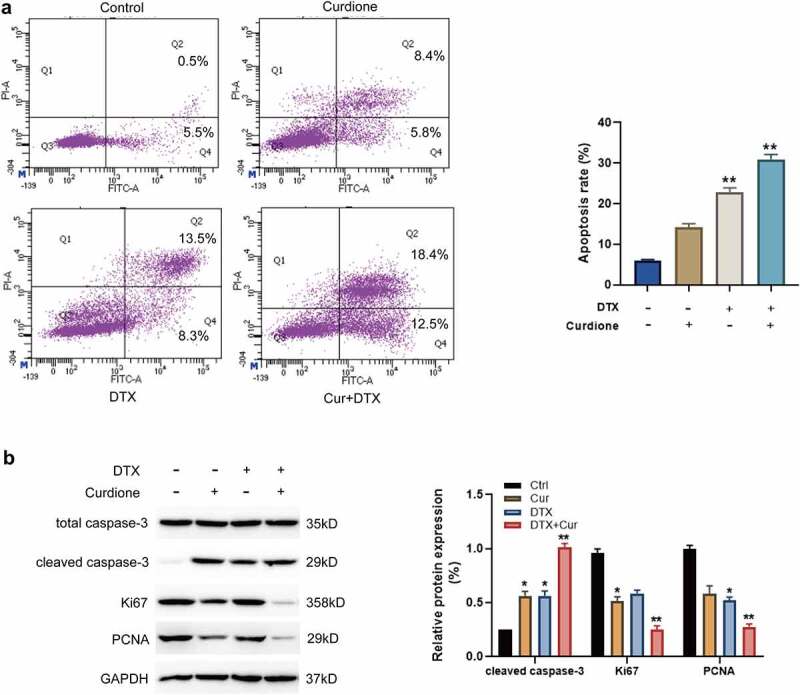
Curdione combined with DTX enhanced pro-apoptotic effects in MDA-MB-468 cells. (a) cell apoptosis in MDA-MB-468 cells with Curdione and/or DTX treatment was analyzed using flow cytometry. The ratios of apoptosis were counted. (b) apoptosis-associated and proliferation-associated proteins (cleaved caspase-3, caspase-3, Ki67, PCNA) were detected in MDA-MB-468 with Curdione and/or DTX treatment. The expression level was quantified by Image J. *p < 0.05, **p < 0.01 versus the control group

Next, we explored the combined effects of Curdione and DTX on MDA-MB-468 cell apoptosis. The FACS results showed that Curdione or DTX alone treatment caused a mild cell apoptosis, while Curdione combined with DTX significantly facilitated cell apoptosis as shown in (Figure 2(a,b)). It is known that cleaved caspase-3 is one of the canonical indicators of cell apoptosis [20]. Ki67 and proliferating cell nuclear antigen (PCNA) are the common proliferative markers in tumor growth. Additionally, we detected these apoptosis and proliferation-related proteins such as cleaved-caspase-3, Ki67, and PCNA. As shown in (Figure 2(c,d)), the cleaved caspase-3 expression was a bit increased with Curdione treatment alone or DTX treatment alone and the increased cleaved caspase-3 was intensified with the combined treatment of Curdione and DTX. Meanwhile, the levels of Ki67 and PCNA were slightly decreased in Curdione-treated cells or DTX-treated cells. The decreased effects were enhanced by the combination of Curdione and DTX. These data suggest that treatment of Curdione and DTX could enhanced the pro-apoptosis effect of DTX on MDA-MB-468 by up-regulating cleaved-caspase-3 protein expression.

### The effects of Curdione and DTX treatment on cellular ROS levels in MDA-MB-468

**Figure 3.**
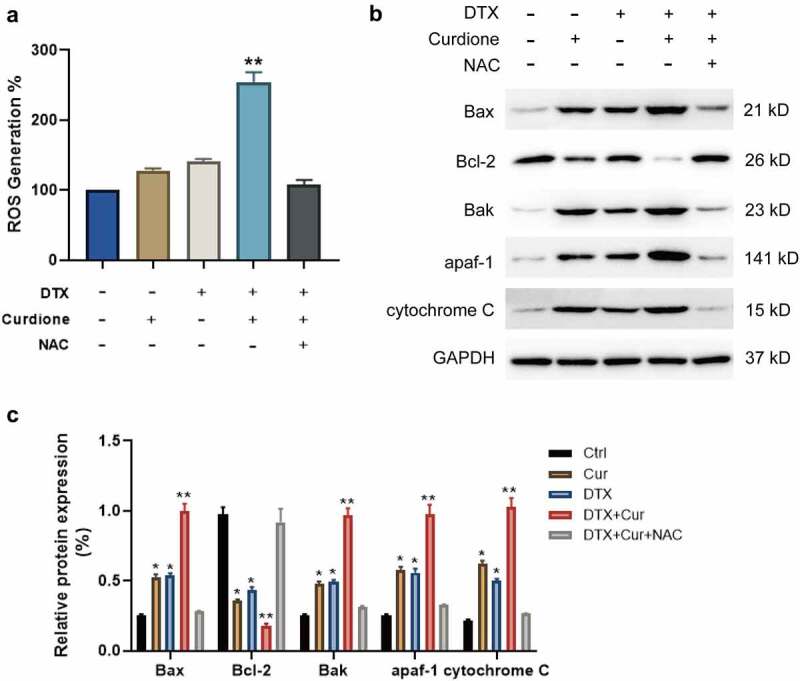
Co-treatment of Curdione and DTX enhanced the cellular ROS production in MDA-MB-468. (a) intracellular ROS production in MDA-MB-468 cells with Curdione, DTX, Curdione and DTX, Curdione and DTX plus NAC (5 mM) treatment was detected using a DCF-DA assay. ROS production was quantitated. (b) the intrinsic apoptosis related proteins were examined in MDA-MB-468 cells with Curdione, DTX, Curdione and DTX, Curdione and DTX plus NAC (5 mM) treatment. (c) the relative protein expression of these related proteins was quantified by Image J. *p < 0.05, **p < 0.01

To explore whether Curdione and DTX treatment promotes cell apoptosis of MDA-MB-468 by inducing ROS generation, we treated cells using the ROS inhibitor NAC. The generation of intracellular ROS in Curdione or DTX-treated cells, Curdione and DTX treated cells, Curdione and DTX plus NAC treated cells. We observed that Curdione alone or DTX alone could induce ROS generation slightly while combined treatment of Curdione and DTX significantly promoted ROS accumulation (Figure 3(a)). Additionally, NAC treatment reduced the ROS amount induced by combined treatment (Figure 3(a)). What’s more, we also observed that the endogenous apoptosis markers including Bax, Bak, apaf-1 and cytochrome c were foldly increased with combined treatment of Curdione and DTX compared to the Curdione or DTX alone treatment while the expression of Bcl-2 was double down-regulated (Figure 3(b,c)). Moreover, NAC treatment could reverse these effects. Taken together, the results indicate that Curdione and DTX treatment could enhanced DTX-induced effects by promoting the ROS-regulated endogenous apoptosis pathway in TNBC cells.

### The alterations of MAPKs and PI3K/Akt signaling pathways

**Figure 4.**
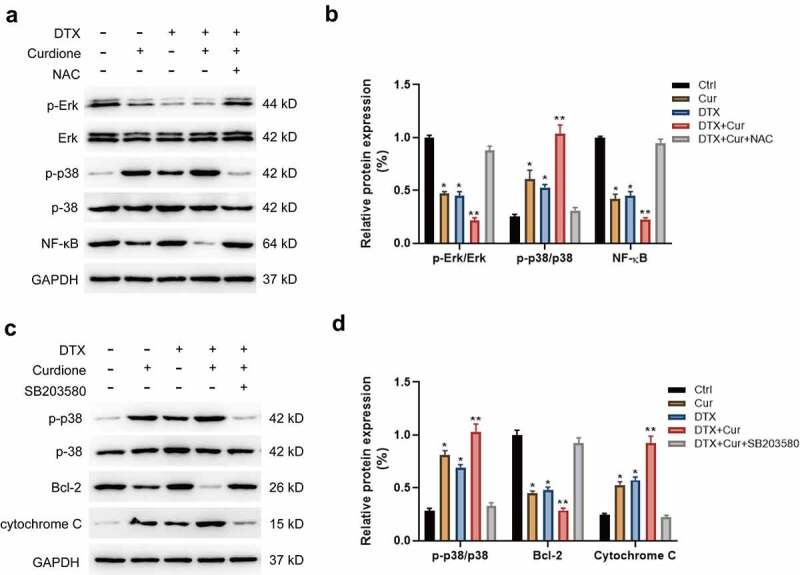
Co-treatment of Curdione and DTX affects MAPK signaling pathway in MDA-MB-468 cells. (a) MAPK signaling pathway related proteins (NF-κB, p38, p-p38, Erk1/2, p-Erk1/2) were detected in MDA-MB-468 cells by western blot. cells were treated with Curdione, DTX, Curdione and DTX, Curdione and DTX plus NAC. (b) The relative protein expression of these related proteins was determined by Image J software. (c) the expression levels of p38, p-p38, Bcl-2, cytochrome c were detected with the treatment of Curdione, DTX, Curdione and DTX, Curdione and DTX plus SB203580 (p38 inhibitor). GAPDH was used as an internal control. (d) the relative protein expression of these related proteins was determined by Image J. *p < 0.05, **p < 0.01

Recent studies have reported that curcuminoids play anticancer roles in multiple solid tumors, of which the underlying mechanism was associated with several signaling pathways such as the nuclear factor κB (NF-κB), MAPK, PI3K/AKT and so on. To clarify the mechanism of the combined treatment of Curdione and DTX enhancing the anti-proliferative and pro-apoptotic effects in TNBC cells, we detected the changes of MAPK and PI3K/Akt signaling pathways. Initially, we found that the combined treatment of Curdione and DTX on MDA-MB-468 obviously activated p38 by increasing the phosphorylated p38 level while decreased NF-κB expression and the phosphorylated Erk1/2 level compared to the single treatment. And the alternation in these proteins could be reversed by co-treatment with NAC (Figure 4(a,b)), suggesting that ROS inhibition attenuated co-treatment-induced effects via MAPKs signals. Moreover, we validated the results by using the p38 inhibitor SB203580 as shown in (Figure 4(c,d)). Pre-treatment of MDA-MB-468 with SB203580 (p38 inhibitor) suppressed the activated MAPK/p38 pathway caused by combined treatment of Curdione and DTX, accompanied by reduced cytochrome c expression and increased Bcl-2 expression (Figure 4(b)). These data suggest that Curdione and DTX treatment promoted ROS-mediated apoptosis of MDA-MB-468 via the MAPK/p38 signaling pathway.

**Figure 5.**
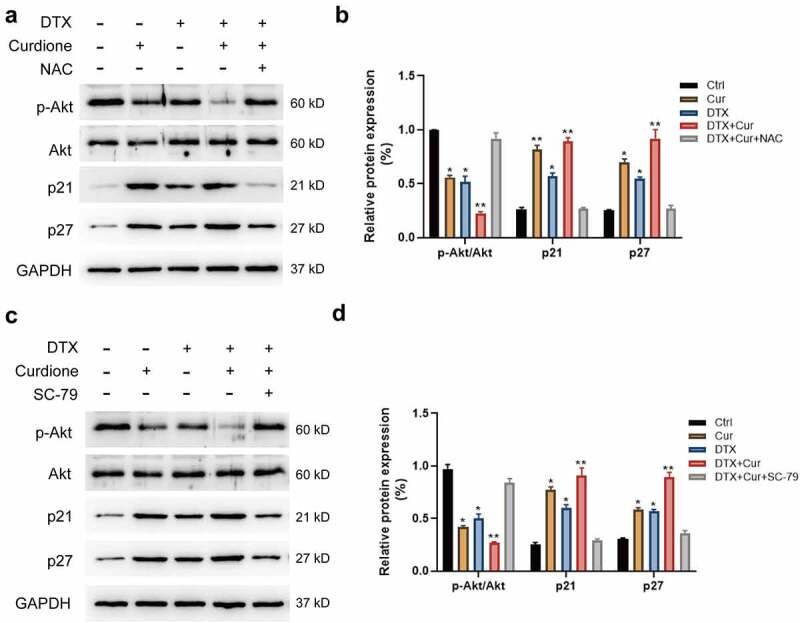
Co-treatment of Curdione and DTX affects PI3K/Akt/p21 signaling in MDA-MB-468 cells. PI3K/Akt signaling pathway related proteins (Akt, p-Akt, p21, p27) were detected in MDA-MB-468 cells by western blot. Cells were treated with Curdione, DTX, Curdione and DTX, Curdione and DTX plus NAC. (b) The relative protein expression of these related proteins was determined by Image J. (c) Expression levels of Akt, p-Akt, p21, p27 were detected with the treatment of Curdione, DTX, Curdione and DTX, Curdione and DTX plus SC-79 (Akt activator). GAPDH was used as an internal control. (d) The relative protein expression of these related proteins was determined by Image J.*p < 0.05, **p < 0.01

Next, we detected whether the PI3K/Akt signaling pathway was altered in MDA-MB-468 cellls with co-treatment of Curdione and DTX. We found that the combined treatment of Curdione and DTX intensified the decreased Akt phosphorylation and CDK1/2 level, and aggravated the increased expression of p21 and p27 compared to the single treatment (Figure 5(a,b)). The alterations caused by co-treatment could be significantly reversed by pre-treatment of NAC (Figure 5(a,b)), indicating that ROS suppression could weaken the synergistic effects caused by co-treatment on MDA-MB-468 through Akt signaling pathway. We also validated the above findings by using SC-79 (Akt activator). As shown in (Figure 5(c,d)), the decreased phosphorylated Akt led to by co-treatment was reversed by the addition of SC-79, along with the increased p21 and p27 levels. The results suggest that combined treatment of Curdione and DTX suppressed cell proliferation by inhibiting PI3K/Akt signaling pathway.

Above all, these results suggest that both MAPK and PI3K/Akt pathways are involved in the combined treatment of Curdione and DTX on MDA-MB-468. Curdione and DTX combination enhanced DTX-induced apoptosis by promoting the ROS production.

## Discussion

DTX has been reported to be one of the antineoplastic agents used for several solid tumors including lung, gastric and breast cancer [21,22]. Nevertheless, as an antineoplastic drug, it is not satisfactory for the application of DTX in TNBC therapy. Researchers have made efforts to find potential DTX chemosensitisers to improve therapeutic effectiveness, which could enhance tumor cells sensitivities and reduce the undesirable adverse effects [23,24]. Recent studies have shown that traditional Chinese medicine (TCM) and a series of compounds extracted from TCM has anti-tumor activities along with hepatoprotective effects [25–27], suggesting that TCM or its extracted compounds would be a better choice as the chemosensitzer. Increasing studies indicated that curcumol could be used as a chemopreventive adjuvant molecule used to enhance the efficacy of chemotherapy drugs [28–30]. For example, it is reported that curcumol potentiates celecoxib-induced growth inhibition and apoptosis in human NSCLC [31]. In this study, it is the first time to identify the synergistical effect of combined treatment of Curdione and DTX in DTX-sensitive MDA-MB-468 cells, which has clinical implications for Curdione as a potential chemosensitzer to synergistically enhance the anticancer efficacy of DTX in TNBC treatment.

Curdione is one of the key components of *Curcuma zedoary*, also known as *Ezhu* is a traditional Chinese medicine [12]. The other components of curcuminoids have been found to inhibit proliferation and induce apoptosis to play anticancer properties in tumors by MAPK, PI3K/Akt, NF-κB pathways [32,33]. For instance, in human nasopharyngeal carcinoma CNE-2 cells, curcumol induces cell cycle arrest and apoptosis by inhibiting IGF-1 R/PI3K/Akt signaling pathway [34]. In colorectal cancer cells LoVo, curcumol inhibits growth and induces apoptosis via IGF-1 R and p38 MAPK pathway [35]. In human non-small lung cancer (NSCLC) cells, curcumol induces apoptosis via NF-κB activation and MAPK and PI3K/AKT signaling pathway [31]. In the present study, we found that combined treatment of Curdione and DTX synergistically altered the protein expressions of MAPKs and PI3K/Akt related signaling pathways. The rescue experiments validated that p38 inhibitor (SB203580) or the Akt activator (SC-79) could prevent the co-treatment-induced apoptosis of MDA-MB-468. NF-κB, PTEN/PI3K/AKT/mTOR, JAK/STAT and receptor tyrosine kinases are implicated in TNBC chemoresistance and progression [36–39]. Consistently, our results demonstrated that the combined strategy of Curdione and DTX synergistically enhanced the anticancer effects in MDA-MB-468 cells via the MAPKs and PI3K/Akt signaling pathways.

Reactive oxygen species (ROS) was found to be used not only as a therapeutic target, but also as a motivator to activate release of antitumor drugs, achieving enhanced efficacy through the combination of chemotherapy and medicine [40,41]. Two studies have reported that curcumol could induce ROS generation: One study reported that curcumol induced reactive oxygen species (ROS) generation in LoVo cells, and ROS scavenger N-acetylcysteine (NAC) could significantly reverse the curcumol-induced cell growth inhibition [42]. The other study showed that the ROS level was increased after the gastric cancer cells were treated with curcumol [43]. Multiple studies have demonstrated that drugs induce cell apoptosis was usually along with the increase of ROS generation [44–46] .In the present study, our data revealed that the combined treatment of Curdione with DTX significantly increased the intracellular accumulation of ROS in contrast to the single treatment. Moreover, NAC treatment could antagonize the increase of ROS induced by co-treatment on MDA-MB-468. Furthermore, we also detected the excessive ROS generation caused intrinsic apoptosis indicators [47] such as cytochrome c, apaf-1 were remarkably increased in MDA-MB-468 induced by the combined treatment of Curdione and DTX in comparison with the single treatment. NAC treatment could suppressed the enhanced intrinsic apoptosis caused by the co-treatment. Taken together, the combined treatment of Curdione and DTX might enhance the DTX-induced effects in MDA-MB-468 cells by increasing intracellular ROS.

Due to the specific characteristics such as negative expression of ER, PR, and HER2, TNBC has limited therapeutic strategies [6,37]. To make matters worse, TNBC is classified into four different subtypes refined by Lehmann et al [5]. These subtypes display varying levels of chemoresistance, which increased the difficulty of treatment. In the experimental process, we initially used MDA-MB-468 and MDA-MB-231 cell lines together. We found that with DTX treatment MDA-MB-231 was more prone to chemotherapy resistance than MDA-MB-468. It was interesting. Considering MDA-MB-468 corresponds to the basel-like 1 subtype, MDA-MB-231 corresponds to the mesenchymal-stem-like subtype, we assume that different subtypes have different sensitivity to combinative treatment strategies as previous reports [37]. Thus we investigated the administration of these two cell lines as two subjects.

However, there are some limitations to our study. Firstly, we did not select more cell lines corresponding to other subtypes to verify the synergistic efficacy of combined treatment of Curdione and DTX in TNBC. Secondly, It is better to perform more functional mechanistic data to support our findings in the further study. The in vivo animal experiments should also be conducted and validated in the future study.

## Conclusions

**Figure 6.**
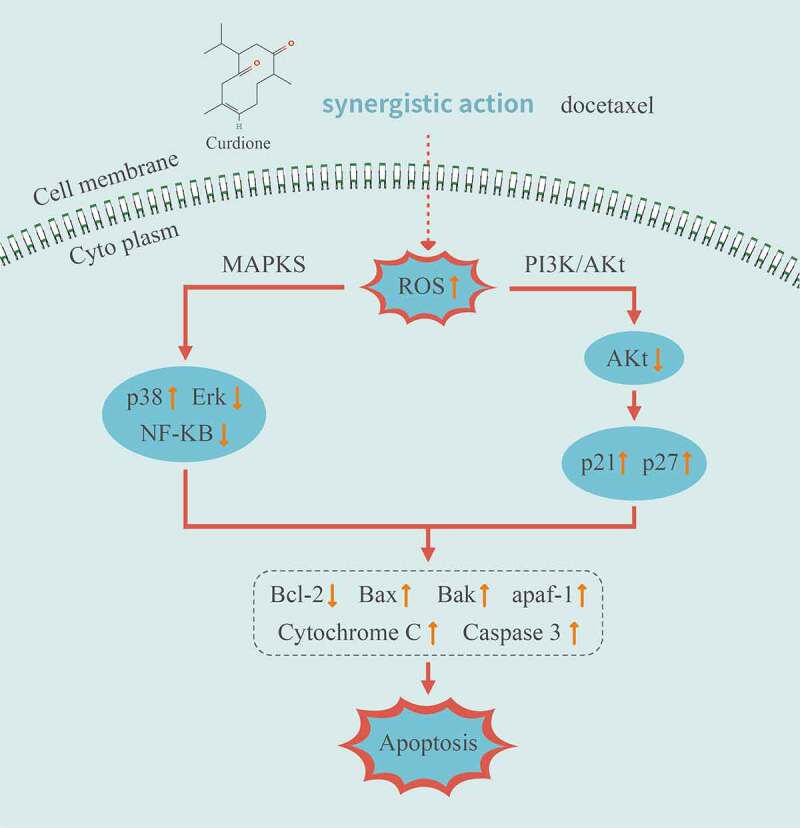
Illustration of the related mechanisms of Curdione combined with DTX effects on the signaling pathways and cellular functions in TNBC cells MDA-MB-468

To sum up, we demonstrated a molecular mechanism of the synergistic effects of Curdione and DTX in TNBC cells MDA-MB-468 (illustrated in Figure 6). The combined strategy of Curdione and DTX exerted cooperative anticancer effects by triggering ROS-induced intrinsic apoptosis via the MAPKs and PI3K/Akt signaling pathways. It is predictable that Curdione might be a promising pharmacological effect enhancer in DTX-treated TNBC treatment.

## Highlights

1. Combined treatment of Curdione and docetaxel has the synergistic effects on MDA-MB-468 cell proliferation suppression and cell apoptosis induction.
2. Combinative treatment of Curdione and DTX synergistically potentiated MDA-MB-468 cell apoptosis by triggering reactive oxygen species (ROS) generation.
3. Combination of Curdione and docetaxel enhanced the chemotherapeutic efficacy on MDA-MB-468 via MAPKs and PI3K/Akt signaling pathways.
